# The Influence of Health Literacy and Social Support on Loneliness Among Patients With Severe Mental Illness in Rural Southwest China

**DOI:** 10.3389/fpsyg.2021.564666

**Published:** 2021-09-08

**Authors:** Yan Liu, Hongdao Meng, Kyaien O Conner, Mutian Qiao, Danping Liu

**Affiliations:** ^1^Department of Health Behavioral and Social Medicine, West China School of Public Health and West China Fourth Hospital, Sichuan University, Chengdu, China; ^2^Department of Academic Affairs, West China School of Medicine/West China Hospital, Sichuan University, Chengdu, China; ^3^School of Aging Studies, College of Behavioral and Community Sciences, University of South Florida, Tampa, FL, United States; ^4^Department of Mental Health Law & Policy, College of Behavioral & Community Sciences, University of South Florida, Tampa, FL, United States

**Keywords:** loneliness, health literacy, social support, severe mental illness, rural, China

## Abstract

Loneliness is an important risk factor for poor health outcomes among adults, especially among those with severe mental illnesses (SMIs). Existing research has shown that adults with SMIs often lack health literacy, which contributes to more restricted social networks and low levels of social support. The objective of this cross-sectional study was to examine the influence of health literacy and social support on the loneliness of patients with SMI in rural Southwest China. We recruited 300 patients with SMI in rural Southwest China between December, 2017 to May, 2018 via a multi-stage stratified random sampling approach. We used structural equation modeling (SEM) test the hypothesized relationships among the variables of the 270 patients who completed the survey. Results of the SEM showed that health literacy was both directly and indirectly associated with loneliness, with social support playing a mediating role. These findings suggest psychoeducation for SMI patients, and their informal caregivers, may offer beneficial effects toward reducing loneliness in this vulnerable population. Further, social support is another potential target for intervention development for improving patient outcomes.

## Introduction

Patients with severe mental illness (SMI) are often described as a group of heterogeneous people who suffer from severe psychiatric disorders together with long-term mental disturbances such as schizophrenia, bipolar disorder, schizoaffective disorders, major recurrent depressive disorder and personality disorders (Jansen, 2018). Patients with SMI often require high levels of care and consume many social and health resources available within the psychiatric and social healthcare network (Nguyen et al., 2020). SMIs contribute significantly to the global burden of disease, impacting more than 4% of the adult population worldwide and leading to substantial premature deaths, as patients with SMI die on average one to two decades earlier than the general population (Wong et al., 2020). In China, with a population of 1.4 billion, the lifetime prevalence of SMI was as high as 10.10‰ (Wang et al., 2017). SMIs are disabling, lethal and often require long-term, complex interventions for both SMI patients and their family caregivers (Vigo et al., 2016).

SMI patients may have fewer successful interactions or have limited social networks because deficiencies in certain social and personal functioning skills such as memory deficits and social anhedonia (Barkus and Badcock, 2019). And they are often excluded socially because of societal stigma against individuals with psychiatric symptoms such as depression or paranoia (Schwartz and Gronemann, 2009). In some cases, individuals with SMI might internalize stigma of mental illness. To be specific, they may feel that having psychopathology makes them perform oddly in social situations or that such performance will be embarrassing, then perhaps they they will create negative affect like feeling inferior and steering clear of people because they have adopted beliefs in the larger society that SMI patients are subordinate to everyone else (Prince et al., 2018). All these experience can increasing the likelihood of feeling lonely. Loneliness, has generally been defined as an unpleasant, distressing, and aversive experience or feeling resulting from the discrepancy between one’s actual versus expected interpersonal relationships (Tharayil, 2007). People who suffer from two or three mental disorders were shown to have approximately 21-fold increase in the odds of loneliness, compared to those with no mental disorder (Meltzer et al., 2013). In addition, it has been reported that loneliness might be part of the causal pathway in the development of psychotic experiences (van der Werf et al., 2010). Loneliness has also been associated with a variety of adverse outcomes including increased risk of relapse, psychiatric hospitalization, medical sequelae, and further social disengagement (Gayer-Anderson and Morgan, 2013; Prince et al., 2018). Studies have found that patients’ subjective recovery from psychosis was significantly related to a decrease in loneliness (Roe et al., 2011), and that of adequate health literacy, social support, and social networks may reduce loneliness among SMI patients (Perese and Wolf, 2005).

Health literacy is commonly defined as having the cognitive ability, knowledge and social skills to obtain, process, and use basic health information and services needed to maintain health and make health care decisions (Medicine, 2004). In China, the correct response rate on the Mental Health Knowledge Questionnaire of the general public was 77.0% (Wang et al., 2013). SMI patients generally report low levels of health literacy (Clausen et al., 2016). Low health literacy is associated with being lowly educated, living in suburban sites and cognitive impairment (Yost et al., 2013). Studies of Masi and colleagues suggest that loneliness can be reduced by addressing abnormal cognition and social skills training (Masi et al., 2011). Geboers et al. (2016) found an association between the lack of health literacy and an increase of loneliness among older adults (Geboers et al., 2016). Previous studies also suggest that poor health literacy in older adults was related to their impoverished social networks and may lead to the loss of necessary social support (Yang et al., 2019). The notion that sufficient social support might buffer and alleviate the adverse health consequences of low health literacy among different groups [like smokers (Stewart et al., 2014) and patients with chronic kidney disease (Lora et al., 2011)] has also been tested.

Social support is a multidimensional concept generally referering to the social resources or help that persons perceived and received (Kang et al., 2018), which can be reflected in three aspects: the perception of support, the provision of support and the structure of social networks (Gayer-Anderson and Morgan, 2013). Lack of social support has been shown to be a strong predictor of loneliness in persons with psychotic disorders (Schwartz and Gronemann, 2009; Chrostek et al., 2016). Perese and collegaues (Perese and Wolf, 2005) found that patients with SMI reportedly experience higher levels of loneliness than the general population, which is linked to low levels of social support. It is well-known that social support has been frequently considered an important resource and is an important component of interventions targeted at reducing loneliness (Hawkley and Cacioppo, 2010; Masi et al., 2011). Previous findings have revealed the improvements in older adults’ social support is due to participation in an exercise intervention which, directly predicted reductions in loneliness (McAuley et al., 2000). Social support may mediate the relationship between other factors (like emotional intelligence) and loneliness (Zou, 2014).

Loneliness, also referred to as “subjective sense of social isolation,” may have a salient impact on the emotional wellbeing, social interaction and recovery process among SMI patients (Linz and Sturm, 2013). Given that loneliness in one person can influence loneliness levels in individuals they interact with (Cacioppo et al., 2009), and family members of SMI patients usually experience substantial caregiver burden (Yu et al., 2017), examining strategies to alleviate loneliness in this disadvantaged population who often feel stigmatized, shamed, isolated and who often report lower health literacy and impoverished social support remains important for clinicians, researchers and policymakers. To date, most previous researches have been conducted to investigate factors influencing loneliness (e.g., health literacy and social support) among different populations. However, the importance of health literacy in loneliness is still rarely assessed, and even less evidence is available about the interrelationships and potential mechanisms of health literacy, social support and loneliness among SMI patients.

## Current Study

**FIGURE 1:**
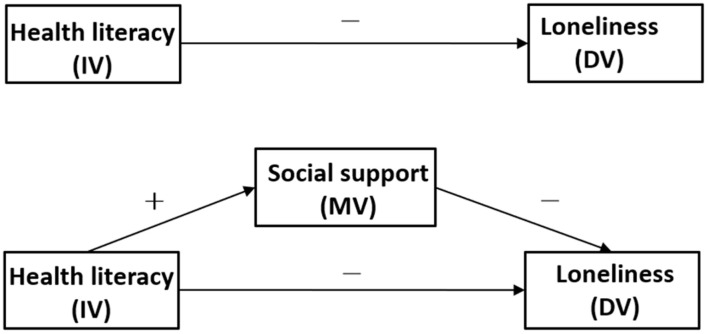
The theoretical model and hypotheses.

The objective of this study was to examine the influence of health literacy and social support on the loneliness of patients with SMI in rural Southwest China. Based on the results of previous studies, we developed a single confirmatory mediator model shown in Figure 1. Specifically, health literacy had a direct positive correlation with social support (hypothesis 1) and had a direct negative correlation with loneliness (hypothesis 2); social support had a direct negative correlation with loneliness (hypothesis 3). In addition, we also hypothesized that the relationship between health literacy and loneliness was mediated by social support (hypothesis 4). This study is the first to explore the influence of health literacy and social support on loneliness among patients with SMI in rural China.

## Materials and Methods

### Participants and Procedure

This cross-sectional research was conducted among SMI patients in Sichuan province, Southwest China from December, 2017 to May, 2018. The target population was the permanent residents aged 18 years or older who had been diagnosed with SMI.

A multi-stage stratified random sampling approach was used to acquire the sample (Pan et al., 2018). In the first stage, we randomly chose a city in Sichuan province. In the second stage, we randomly selected a rural district in the city. In the third stage, ten townships were randomly selected from the rural district. In the fourth stage, we randomly selected 30 SMI patients from the database of SMI patients established by each township hospital. Township hospitals are required to receive patients with severe mental illness clearly diagnosed by mental health institutions among permanent residents within their jurisdiction, input patients’ information into the database, and provide intervention, follow-up and physical examination services for them. Acceptance into services at township hospitals required meeting relevant “diagnostic criteria” in the “Classification of Mental and Behavioral Disorders” section (ICD-10, F00-F99) of the International Classification of Diseases (WHO, 1992) for severe mental illness, which included but is not limited to a diagnosis of Schizophrenia; schizoaffective disorder, paranoid psychosis, bipolar disorder, epilepsy with psychotic features (Hyman et al., 2011). Based on census information from township hospitals, a close approximation of the diagnositc breakdown of the participants in this study as follows: collectively 83.0% were diagnosed with schizophrenia, 14.4% with mental retardation with psychotic features, 1.9% with epilepsy with psychotic features, and 0.7% with other psychiatric disorders (bipolar disorder, major recurrent depressive), 30.4% was taking antipsychotic medications. Patients were excluded if deemed by their clinician to have insufficient capacity to consent and respond at the time of assessment. A total of 300 eligible SMI patients were interviewed face-to-face by professionally trained investigators and 270 SMI patients completed the questionnaire (for a valid response rate of 90.0%).

### Ethical Considerations

The study protocol was approved by the Institutional Review Board of Sichuan University (Project identification code: H171260). Informed consent was obtained from each SMI patients following a detailed explanation about the purpose of this research.

### Measures

SMI patients’ socio-demographics, health literacy, social support and loneliness information were collected from questionnaires.

### Socio-Demographics

Socio-demographics included gender, age, marital status, education level and per capita annual income of the household.

### Health Literacy

Health literacy was assessed by the subscale from the Questionnaire of Mental Health Work issued by the Chinese Ministry of Health: the Mental Health Knowledge Questionnaire (MHKQ) (Liang et al., 2011; Wang et al., 2013). The questionnaire used in this study included 16 items, such as “Mental disorders are caused by incorrect thinking” and “Psychological problems can occur at almost any age,” which are about basic knowledge of mental health issues. One point is given for each correct answer, with incorrect or unknown responses receiving 0 points. The total scores range from 0 to 16, with higher scores representing better health literacy. This scale can be used to monitor changes in mental health literacy over time as the mental health promotion activities envisaged. Previous studies have shown that this scale is a validated mental health literacy instrument (Liang et al., 2011). Wang et al. assessed the reliability and validity of the final version of this scale in Chinese adult community members and found that the internal consistency of the 16-item MHKQ was 0.59. In this study, Cronbach’s alpha of the scale was 0.71, KOM value of the scale was 0.71.

### Social Support

Social support was measured using the Social Support Rating Scale (SSRS) (Xiao ShuiYuan, 1987), designed by Xiao and colleagues. The SSRS is a self-report scale including ten items and three domains: Objective support, refering to an individual’s social networks, and the instrumental and emotional support they have actually received in the past. Subjective support, refering to the emotional support experienced by the individual, that is, the emotional experience and satisfaction of being respected, supported and understood in the society. Social support utilization, refering to the extent to which support is utilized. Responses were provided using a 4-point Likert scale, with total scale scores ranging from 12 to 66, with higher scores indicating stronger social support. In the current study, the total score was divided into three levels: low (12–22), moderate (23–44), and high social support (45–66). The SSRS has been shown to have good reliability and validity in Chinese patients with schizophrenia and is considered to be an easily understandable instrument to assess social support in Chinese populations (Tao et al., 2012). In the current study, Cronbach’s alpha of the scale was 0.71, KOM value of the scale was 0.70.

### Loneliness

Loneliness was measured by the University of California at Los Angeles loneliness scale (UCLA) (Russell, 1996). The scale included 20 items pertaining to loneliness, with 11 positive and 9 negative items, such as “How often do you feel that you lack companionship?” and “How often do you feel that you are “in tune” with the people around you?” to reflect how people sometimes feel. Each of the 20 items is rated on a scale of 1 (never), 2 (rarely), 3 (sometimes), and 4 (always). The range of possible scores was 20–80, with higher scores signifying greater loneliness. The total score has been divided into three levels: mild loneliness (20–34), moderate loneliness (35–49), and severe loneliness (50–80). The Chinese version of the scale, translated by Wang et al., has demonstrated adequate reliability and validity in different populations including populations with schizophrenia (Wang, 1993; Schwartz and Gronemann, 2009). In this sample, Cronbach alpha was 0.79, KOM value of the scale was 0.86.

### Data Analytical Plan

Data were entered using the Epidata3.1 database. Statistical analyses were conducted using SPSS version 20.0 (IBM, Chicago, IL, United States) for the descriptive analysis, linear regression analysis and Pearson correlation coefficient, and Mplus version 7.0 (Los Angeles, CA, United States) for the covariance structure analysis. The level of statistically significant differences was set at *p* < 0.05.

For the first step, descriptive statistics (frequencies, percentages, means, and standard deviations) were calculated for socio-demographics and the key study variables, followed by Pearson correlations among these variables. Subsequently, a linear regression model was also used to analyze the influence of socio-demographics, in addition to health literacy and social support on loneliness. Ultimately, a structural equation model (SEM) was employed to further test the hypothesized relationships among health literacy, social support and loneliness of SMI patients (Baron and Kenny, 1986; Gunzler et al., 2013).

**FIGURE 2:**
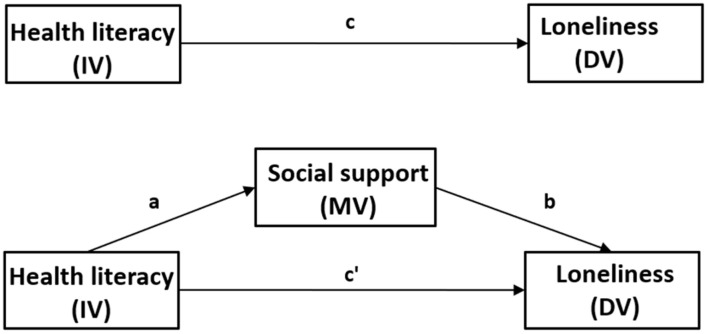
Schematic model with social support as the mediator in the relationship between health literacy and loneliness. IV, independent variable (predictor); DV, dependent variable; MV, mediator variable. Path (c) represents the total effect of health literacy on loneliness (without considering the mediating effect of social support). Path (a) refers to the direct impact of health literacy on social support. Path (b) depicts the direct impact of social support on loneliness after controlling for health literacy. Path (c’) represents the direct impact of health literacy on loneliness after controlling for social support. The indirect effect of health literacy on loneliness via social support is calculated as the product of the direct effects of health literacy on social support and social support on loneliness (a × b).

Using Baron and Kenny (1986) guidelines, to establish mediation, following steps need to be followed: (1) the independent variable (IV) predicts the presumed mediator variable (MV); (2) the MV predicts the dependent variable (DV), controlling for the IV; (3) after controlling for the effects of the MV, a previously significant relationship between the IV and the DV becomes non-significant (full or perfect mediation) or weaker (partial mediation). Thus, following the procedure proposed by Baron and Kenny, the analyses of the role of social support in mediating the relationship between health literacy and loneliness were planned in several stages (see Figure 2).

## Results

### Sociodemographic Characteristics of the Participants

Socio-demographic characteristics of the 270 SMI patients are displayed in Table 1. Of the total sample, the mean (SD) age of the total SMI patients was 49.7 (13.4) years and 44.1% were females. Almost a third were unmarried (31.1%) and two third had elementary school education or less (62.2%). About half of the SMI patients had a per capita annual household income of less than $750 (48.5%).

**TABLE 1:** Socio-demographic characteristics of the patients with severe mental illness (*n* = 270).

Socio-demographic characteristics	*n*	%
**Gender**		
Female	119	44.1
Male	151	55.9
**Age, group**		
18∼39	57	21.1
40∼64	164	60.7
≥65	49	18.1
**Marital status**		
Single	84	31.1
Married	137	50.7
Divorced or widowed	49	18.1
**Education level**		
Less than elementary school	63	23.3
Elementary school	105	38.9
Middle school	69	25.6
High school and above	33	12.2
**Per capita annual income of the household ($)**		
<750	131	48.5
750∼1499	56	20.7
≥1500	83	30.7

### Descriptive Analysis of Study Variables

**FIGURE 3:**
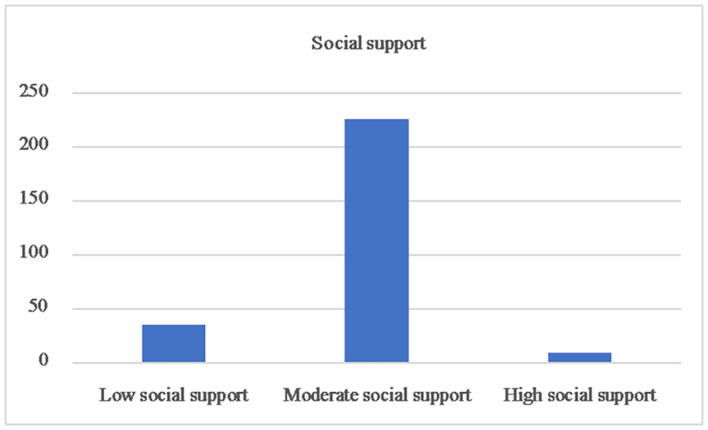
Social support of the patients with severe mental illnes.

**FIGURE 4:**
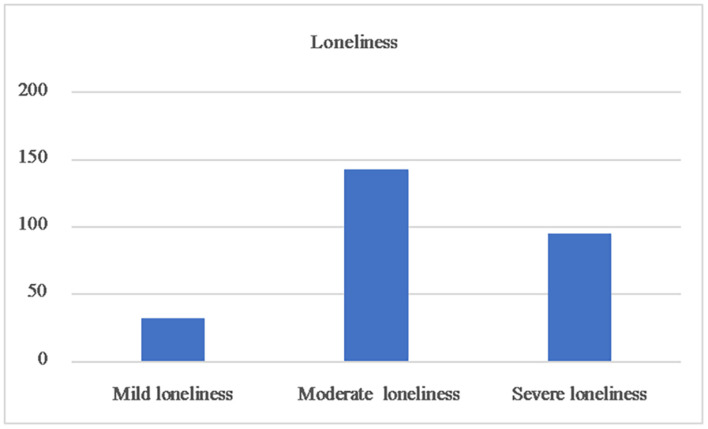
Loneliness of the patients with severe mental illnes.

Table 2 shows the mean scores of the key variables for the entire sample. For health literacy, the mean score was 10.1 ± 1.9 and the computed score ranged from 4 to 16. The proportion of respondents who answered each item correctly ranged from 26.3 to 97.4%; the mean correct response rate for the 16 items was 63.1%. As is shown in Table 2 and Figure 3, the mean score for social support was 30.2 ± 7.2. Based on the score, 13.0% (35), 83.7% (226), and 3.3% (9) of SMI patients had low, moderate, and high social support, respectively. As is shown in Table 3 and Figure 4, the mean score for loneliness was 45.8 ± 8.0, with 11.8% (32) of SMI patients experiencing mild loneliness, 53.0% (143) and 35.2% (95) of SMI patients experiencing moderate and severe loneliness, respectively.

**TABLE 2:** Description of health literacy, social support and loneliness scores (*n* = 270).

**Contents**	**Range**	**Mean (SD)**
Health literacy	0–16	10.11.9
Social support	12–66	30.27.2
Objective support	1–22	6.72.1
Subjective support	8–32	17.94.8
Support utilization	3–12	5.62.1
Loneliness	20–80	45.88.0

**TABLE 3:** Correlation coefficients among study variables.

Variables	(1)	(2)	(3)
(1) Health literacy			
(2) Social support	0.328**		
(3) Loneliness	−0.507**	−0.406**	

**p < 0.01.

### Correlations of the Study Variable

The Pearson’s correlations for the study variables are presented in Table 3. Health literacy was positively correlated with social support and negatively correlated with loneliness. Social support was negatively correlated with loneliness.

### Regression Analysis of Study Variable

We used loneliness as the dependent variable and socio-demographic variables, health literacy and social support as independent variables in the linear regression model. Table 4 showed three socio-demographic factors in addition to health literacy and social support were significantly correlated with loneliness: gender, age, and marital status. Male SMI patients were more likely to endorse loneliness (β = 0.162, *p* = 0.005). SMI patients aged 40 to 64 (β = −0.137, *p* = 0.044) were less likely to endorse loneliness when compared with those aged 18 to 39. Married SMI patients (β = −0.249, *p* < 0.001) were less likely to be lonely when compared with single patients.

**TABLE 4:** Linear regression of factors associated with loneliness.

Factors	Unstandardized coefficients		Standardized coefficients	*t*	*p*-value	95% CI for β
	β	SE	β			
Constant	75.880	2.929		25.902	< 0.001	(70.111, 81.649)
**Gender (ref: Female)**						
Male	2.615	0.925	0.162	2.826	0.005	(0.792, 4.437)
**Age (ref:18∼39)**						
40∼64	–2.251	1.114	–0.137	–2.020	0.044	(−4.445, −0.057)
≥65	–2.409	1.448	–0.116	–1.664	0.097	(−5.260, 0.442)
**Marital status (ref: Single)**						
Married	–4.319	1.154	–0.249	–3.743	< 0.001	(−6.591, −2.047)
Divorced or widowed	–1.427	1.149	–0.069	–1.242	0.216	(−3.690, 0.836)
**Education level (ref: Less than elementary school)**						
Elementary school	0.592	1.071	0.036	0.552	0.581	(−1.518, 2.701)
Middle school	1.143	1.204	0.062	0.950	0.343	(−1.227, 3.514)
High school and above	–1.746	1.518	–0.071	–1.151	0.251	(−4.735, 1.243)
**Per capita annual income of household (ref:<750,$)**						
750∼1,499	1.969	1.053	0.099	1.870	0.063	(−0.105, 4.044)
≥1,500	–0.757	0.951	–0.044	–0.796	0.427	(−2.630, 1.116)
Health literacy	–1.750	0.226	–0.415	–7.738	< 0.001	(−2.196, −1.305)
Social support	–0.365	0.064	–0.326	–5.724	< 0.001	(−0.491, −0.239)

*R^2^ = 0.36, F = 13.77, *p* < 0.001.*

### Test of Study Model

To examine the relationship between all of the variables, a structural equation model was estimated from the results of the linear regression analysis. Path analysis was further employed to test the hypothesized full model after controlling for the socio-demographics. With the addition of socio-demographics as covariates, the arrow direction among the core variables in the SEM remained unchanged and the corresponding coefficients did not change significantly. Thus, the socio-demographics were not confounding factors and the influence path of socio-demographics was not shown in the final model.

**FIGURE 5:**
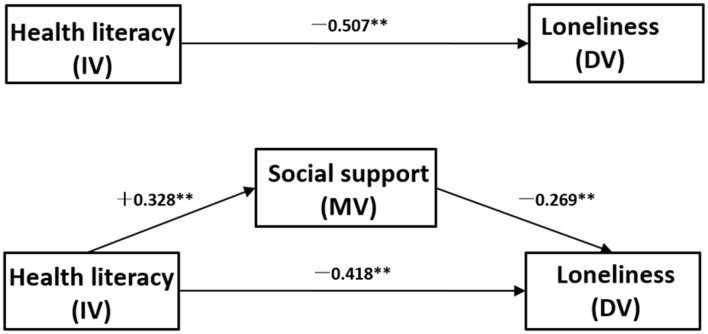
The final model and standardized model paths.

The SEM used bootstrap maximum likelihood estimation. The fit between the current data and the hypothesized model was assessed through several indicators: the comparative fit index (CFI) and the Tucker-Lewis index (TLI) of 0.97 or above; the root mean square error of approximation (RMSEA) and the standardized root mean square residual (SRMR) less than or equal to 0.05 and a x^2^/df of < 3, indicated an good model fit (Schermelleh-Engel et al., 2003). Figure 5 shows the final model where all paths were statistically significant and the model fit the data well: CFI = 1.00; TLI = 1.00; RMSEA = 0.00; SRMR = 0.00 and X^2^/df < 3.00.

Bootstrap with 2000 replications using maximum-likelihood estimation was employed for each path. The estimates for direct, indirect and total effects with bias-corrected 95% CI are shown in Table 5. In these analyses, effect coefficients were substantially significant if the 95% CI does not include 0 (Hayes, 2009). The results showed that all path coefficients were substantially significant: Health literacy negativly predicts loneliness (c) [β = −0.507, 95% CI: (−0.604) to (−0.388)] and positively predicts social support (a) (β = 0.328, 95% CI: 0.201–0.451). Social support negativly predicts loneliness (b) [β = −0.269, 95% CI: (−0.379) to (−0.138)], controlling for health literacy. After controlling for the effects of social support, a previously significant relationship between health literacy and loneliness becomes weaker (c’) [β = −0.418, 95% CI: (−0.523) to (−0.296)]. Based on the above, health literacy negativly indirectly predicts loneliness through the partial mediating role of social support (a × b) [β = −0.089, 95%CI: (−0.146) to (−0.046)].

**TABLE 5:** Direct, indirect and total effects and 95% confidence intervals for the final model.

Model pathways	Estimated effect	95% CI
**Total effects**		
Loneliness <— Health literacy	–0.507	(−0.604) to (−0.388)
Social support <— Health literacy	0.328	0.201 to 0.451
Loneliness <— Social support	–0.269	(−0.379) to (−0.138)
**Direct effects**		
Loneliness <— Health literacy	–0.418	(−0.523) to (−0.296)
Social support <— Health literacy	0.328	0.201 − 0.451
Loneliness <— Social support	–0.269	(−0.379) to (−0.138)
**Indirect effects**		
Loneliness <— Health literacy	–0.089	(−0.146) to (−0.046)

*CI, confidence interval.*

## Discussion

The current study examines the effect of health literacy and social support on loneliness among a sample of patients with SMI in China. In 270 SMI patients, 88.2% of SMI patients experienced moderate or more severe loneliness, which is significantly higher than Chinese older adults (19.6%) (Yang, 2020) and adults with a diagnosis of psychosis in Australia (80%) (Stain et al., 2012). People with SMI are often excluded socially because of their psychiatric symptoms (such as depression and paranoia) and the lack of social acceptance. In addition, patients themselves may feel inferior and actively isolate or create distance with other individuals by internalizing stigma (Prince et al., 2018). Thus, the experience of loneliness is particularly prevalent among patients with SMI, due to their deficiencies in certain physical, psychological, and social functioning skills (Schwartz and Gronemann, 2009).

The results of this research indicated that only 3.3% of the individuals with SMI identified a high level of social support, which was lower than previous surveys of the general population (56.9%) (Jiangfei et al., 2017), indicating that the social support among this group is seriously impoverished. This finding is consist with previous research, which suggests that those with psychosis are more likely to have reduced social networks and limited access to satisfactory social support compared with others (Buchanan, 1995; Macdonald et al., 2000). In the Chinese context, social support is based on a strict social relationship network and is maintained through the reciprocate of favors (Kleinman and Kleinman, 1993). A diagnosis of SMI and its onsets were events of “losing face,” which would isolate a family from mutual help so as to lose support (Yang and Kleinman, 2008). Low household income may also exacerbate caregiving burden and lead to poorer social support (Chiou et al., 2009). In current study, 69.2% of participants had a per capita annual income of household less than $1500, lower than the per capita annual income of Chinese rural residents ($2924) and urban residents ($6127) in 2018 (Statictics, 2018). As expected, the model results confirmed the existence of a negative direct association between social support and loneliness among SMI patients which is consistent with the afore mentioned notion that social support acts as a protective factor by decreasing the feelings of loneliness (Institute of Medicine, 2004; Yildirim and Kocabiyik, 2010). The evidence presented by Sheridan and colleagues (Sheridan et al., 2015) and Zhang and colleagues (Zhang et al., 2018) indicate a clear trend that adequate social support and socializing interventions for people enduring mental illness lead to improved social functioning and reduced levels of loneliness. This suggests that to decrease loneliness in SMI patients, enhancing social support should be considered.

The mean overall health literacy (operationalized as the correct response rate on the MHKQ) of SMI patients in this study was 63.1%, which is lower than that of the general public in China (77.0%) (Wang et al., 2013). Cognitive or functional impairments which may accompany the presence of a mental illness, and poverty are established risk factors for low health literacy, which may help to explain this finding (Camann, 2001; Clausen et al., 2016). In addition, 62.2% of the participants in the sample had an elementary school education or less, representing a lower educational level than that of the overall general population in China (33.8%), the rural population in China (45.3%), and the rural population in Sichuan Province (54.7%), as collected from The Sixth National Census (Statictics, 2010). The data indicates that the general education level among sample population in this study was low, which is typically associated with low health literacy (Yost et al., 2013). As hypothesized, the SEM results of the current study suggest that SMI patients with lower health literacy were more likely to endorse loneliness, consistent with the relationship reported by Geboers and colleagues (Geboers et al., 2016). Individuals with low health literacy not only lack knowledge around positive health behaviors or self-help strategies but also have difficulties recognizing their mental health issues and making successful health decisions (Pignone et al., 2005; Kim et al., 2017; Degan et al., 2019b). Further, in China, where personal and social stigma toward mental illness is more prevalent than that in Western countries, individuals with low health literacy who experience SMI may be more likely to hide their plight due to shame and may withdraw from social contacts for fear of rejection (Parikh et al., 1996; Corrigan, 2015; Zhuang et al., 2017). These findings highlight the need to increase overall health literacy of SMI patients and confirm the importance of health literacy in decreasing feelings of loneliness.

The model results suggest that SMI patients’ health literacy is direct positively associated with their social support: the higher the levels of health literacy, the better the levels of social support, which has been supported by previous studies (Lee et al., 2018; Degan et al., 2019a). Improving health literacy can be considered as a possible public health strategy for encouraging help-seeking, those who have better health literacy may be more likely to acquire adequate support and apply their resources to solving their health problems (Suka et al., 2015). The most significant finding of this study was the partial mediating role of social support in the relationship between health literacy and loneliness. Our results suggest that low health literacy is directly related to SMI patients’ loneliness; and it can indirectly affect loneliness through the mediation of social support. Furthermore, effect contrasts indicated that the direct effect of health literacy was statistically significantly greater than that of social support. Thus, to decrease the loneliness level of SMI patients, health literacy can be regarded as an important intervening target.

According to Brijnath et al. (2016), psychoeducation and abnormal cognitive-behavioral therapy for SMI patients may be beneficial toward improving health literacy and ultimately reducing self perceived lonliness. Meanwhile, education and therapy strategies should take into account the understanding and receptivity of SMI patients with low education level. Other possible approaches are to increase patients’ opportunities for social contact, improve their social networks and enhance social support received from family and friends (Lee et al., 2004). However, if members of an individuals social network have inadequate knowledge about mental illness, their support could be unhelpful or even harmful (Griffiths et al., 2011). Therefore, public education campaigns for patients’ proxies or direct caregivers are also needed.

Of note, results of the present study indicated that the gender, age and marital status of SMI patients were associated with loneliness. Males with SMI were more likely to endorse loneliness, which is in line with the previous research (van den Broek, 2017). Men may have less multifaceted social networks resulting in poorer social support (Zhou et al., 2018). In addition, SMI patients who were middle-aged or patients who were married in this study were less likely to be lonely, which is consistent with previous research (Perlman and Peplau, 1984; Luhmann and Hawkley, 2016). One possible explanation may be that being unmarried, not having a partner or living alone, means that needs for emotional or instrumental support are more likely to be unmet, thus increasing the risk of loneliness. Another possible explanation may be that young adults may have higher expectations about their social networks and interactions, while middle-aged adults have more accumulated resources with age, may be more easily satisfied with their social relations or there is greater alignement between their acutal and expected social interactions (Perlman and Peplau, 1984). This suggests that more attention should be paid to male, young and single individuals with SMI with regard to social support and lonliness. The study did not find the relationship between loneliness and educational level of SMI patients, which is consistent with previous research (Ferreira-Alves et al., 2014). Contrary to previous studies (Nicolaisen and Thorsen, 2014), the linear regression results of this study showed that there is no association between SMI patients’ per capita annual income of household and lonliness. Future Studies can further explore the specific impact of income and its related factors on loneliness in SMI patients.

The examination of factors which contribute to loneliness among people with SMI is important. Addressing loneliness might improve general health, and can lead to positive patient centered outcomes (e.g., reduction in behavioral health symptoms, recovery) and reduce negative health outcomes (e.g., re-hospitalization). A limited number of studies have discussed the relationship between health literacy and loneliness and no study, to our knowledge, has addressed these variables in SMI patients. The present study emphasized the role of health literacy and social support as the predictors of loneliness among individuals with SMI. Findings of this study enrich the explanation of the mechanism supporting the association between health literacy and loneliness and can be referenced to ameliorating the loneliness of the individuals with SMI. The main limitations of this study also need to be considered. First, the participants in the present study are representative of individuals with SMI in Southwest China and findings might differ in other populations, urban areas or other countries. Second, the questionnaire in this study was conducted by self-report, and the respondents may have some report bias. Third, most of the scales appear to have low internal consistency scores, low internal consistency of the measures makes it difficult for us to assume that the variance explained is actually due to the measures themselves. And as we did not collect data relating to mental health diagnosis and medication information, it is not clear if our findings might be impacted by these background information of SMI patients. Furthermore, the cross-sectional nature of the data makes it impossible to allow conclusions on causality. Future Longitudinal or experiment studies may be conducted provide more definite information about the causal inference.

## Conclusion

In conclusion, this study aimed to investigate the relationships among health literacy, social support, and loneliness among Chinese individuals with SMI. Results suggest that health litercay and social support exert a significant direct negative influence on loneliness. The results also suggest that social support played a mediating role in the association between health literacy and loneliness. It is likely that the influence of low health literacy on loneliness could be buffered further by adequate social support, highlighting the importance of the identification of interventions targeting health literacy and social support which could ultimately decrease perceptions of lonliness among SMI patients. Psychoeducation and abnormal cognitive-behavioral therapy for SMI patients, education campaigns for patients’ proxies or direct caregivers may be beneficial to this end. For the socio-demographic characteristics, male, youth and single were predictive of higher levels of SMI patients’ loneliness. Future interventions may also target males, young adults and single SMI patients as they were found in the current study to have higher levels of lonliness.

## Data Availability Statement

The original contributions presented in the study are included in the article/Supplementary Material, further inquiries can be directed to the corresponding author/s.

## Ethics Statement

The studies involving human participants were reviewed and approved by Institutional Review Board of Sichuan University. The patients/participants provided their written informed consent to participate in this study.

## Author Contributions

DL conceived the study and prepared the study protocol. YL performed the data analysis and drafted the first manuscript. All authors contributed to content revisions, approved the final version, and agreed to be responsible for all aspects of the current work.

## Conflict of Interest

The authors declare that the research was conducted in the absence of any commercial or financial relationships that could be construed as a potential conflict of interest.

## Publisher’s Note

All claims expressed in this article are solely those of the authors and do not necessarily represent those of their affiliated organizations, or those of the publisher, the editors and the reviewers. Any product that may be evaluated in this article, or claim that may be made by its manufacturer, is not guaranteed or endorsed by the publisher.
