# Supplementary material for: The Influence of Health Literacy and Social Support on Loneliness Among Patients With Severe Mental Illness in Rural Southwest China
Source: Front Psychol. 2021 Sep 8;12:564666. doi: 10.3389/fpsyg.2021.564666 (PMC8455823; doi:10.3389/fpsyg.2021.564666)
Supplement: Supplementary file 1 [file Data_Sheet_1.ZIP › Supplementary File/Questionnaire——Chinese.doc]

**问卷调查表**

**（一）社会人口学特征调查**

1.	您的性别：

1）女	2）男

2.	您的年龄：

1）18-39	2）40-64	3）≥65

3.	您的婚姻状况：

1）未婚	2）已婚	3）离婚或丧偶

4.	您的文化程度：

1）低于小学	2）小学	3）初中	4）高中及以上

5. 您的家庭人均年收入大约是（元）：

1）＜5000	2）5,000-9,999	3）≥10,000

**（二） 健康素养情况调查**

对以下说法，您的看法是什么：

|  | 赞成 | 反对 |
| --- | --- | --- |
| 1. 心理健康是健康的一个组成部分。 | ① | ② |
| 2. 精神疾病就是思想上出了问题。 | ① | ② |
| 3. 很多人都可能存在心理问题，但自己可能意识不到。 | ① | ② |
| 4. 精神疾病都是因为受了刺激。 | ① | ② |
| 5. 心理健康的主要内容包括智力正常、情绪稳定、心情愉快、人际关系融洽、适应能力良好等。 | ① | ② |
| 6. 绝大多数精神疾病是治不好的。 | ① | ② |
| 7. 怀疑有心理问题或精神疾病就要去找心理医生或者精神科医生看病。 | ① | ② |
| 8. 几乎任何年龄阶段的人都有可能出现心理问题。 | ① | ② |
| 9. 精神疾病和心理问题是没有办法预防的。 | ① | ② |
| 10. 即使确诊为严重精神疾病，如精神分裂症，吃一段时间药就可以了，没有必要长期不间断地服药。 | ① | ② |
| 11. 乐观开朗的生活态度、良好的人际关系和健康的生活习惯有助于我们保持心理健康。 | ① | ② |
| 12. 家族中有精神疾病的人，出现心理问题和精神疾病的可能性比一般人更大。 | ① | ② |
| 13. 青少年的心理问题不会影响其学习成绩。 | ① | ② |
| 14. 中老年人出现心理问题和精神疾病的可能性很小。 | ① | ② |
| 15. 有些人的性格不好，比较容易出现心理问题。 | ① | ② |
| 16. 心理压力大，或者遇到重大的事件（如亲人去世），就容易诱发心理问题和精  神障碍。 | ① | ② |

**三）社会支持情况调查**

1. 您有多少关系密切，可以得到支持和帮助的朋友？

1）一个也没有	2）1—2个	3）3-5个	4）6个或 6个以上

2. 近一年来您：

1）远离家人，且独居一室	2）住处经常变动，多数时间和陌生人住在一起

3）和同学、同事或朋友住在一起	4）和家人住在一起

3. 您与邻居：

1）相互之间从不关心，只是点头之交	2）遇到困难可能稍微关心

3）有些邻居都很关心您	4）大多数邻居都很关心您

4. 您与同事：

1）相互之间从不关心，只是点头之交	2）遇到困难可能稍微关心

3）有些同事很关心您	4）大多数同事都很关心您

5.1 您从夫妻（恋人））得到的支持和照顾

1）无	2）极少	3）一般	4）全力支持

5.2 您从父母得到的支持和照顾

1）无	2）极少	3）一般	4）全力支持

5.3 您从儿女得到的支持和照顾

1）无	2）极少	3）一般	4）全力支持

5.4 您从兄弟姐妹得到的支持和照顾

1）无	2）极少	3）一般	4）全力支持

5.5 您从其他成员（如嫂子）得到的支持和照顾

1）无	2）极少	3）一般	4）全力支持

6. 过去，在您遇到急难情况时，曾经得到的经济支持和解决实际问题的帮助的来源有：

1）无任何来源

2）下列来源（可多选）：A.配偶； B.其他家人；C.朋友 D.亲戚；E.同事；F.工作单位；G.党团工会等官方或半官方组织	H.宗教、社会团体等非官方组织；

I.其它（请列出：	）

7. 过去，在您遇到急难情况时，曾经得到的安慰和关心的来源有：

1）无任何来源

2）下列来源（可多选）：A.配偶； B.其他家人；C.朋友 D.亲戚；E.同事；F.工作单位；G.党团工会等官方或半官方组织 H.宗教、社会团体等非官方组织；

I.其它（请列出：	）

8. 您遇到烦恼时的倾诉方式：

1）从不向任何人诉述	2）只向关系极为密切的 1-2 个人诉述

3）如果朋友主动询问您会说出来	4）主动诉述自己的烦恼，以获得支持和理解。

9. 您遇到烦恼时的求助方式：

1）只靠自己，不接受别人帮助	2）很少请求别人帮助

3）有时请求别人帮助	4）有困难时经常向家人、亲友、组织求援

10. 对于团体（如党团组织、宗教组织、工会、学生会等）组织活动，您：

1）从不参加	2）偶尔参加	3）经常参加	4）主动参加并积极活动

**（四）孤独情况调查**

0 从不	1 很少	2 有时	3 经常

| 请根据您日常的感受选择最符合您情况的答案： | 0 | 1 | 2 | 3 |
| --- | --- | --- | --- | --- |
| 1. 您常感到与周围的人关系和谐吗？ |  |  |  |  |
| 2. 您常感到缺少伙伴吗？ |  |  |  |  |
| 3. 您常感到没有人可以信赖吗？ |  |  |  |  |
| 4. 您常感到寂寞吗？ |  |  |  |  |
| 5. 您常感到属于朋友中的一员吗？ |  |  |  |  |
| 6. 您常感到与周围的人有很多共同点吗？ |  |  |  |  |
| 7. 您常感到与任何人都不亲密了吗？ |  |  |  |  |
| 8. 您常感到你的兴趣与想法与周围的人不一样吗？ |  |  |  |  |
| 9. 您常感到想要与人来往、结交朋友吗？ |  |  |  |  |
| 10. 您常感到与人亲近吗？ |  |  |  |  |
| 11. 您常感到被人冷落了吗？ |  |  |  |  |
| 12. 您常感到您与别人来往毫无意义吗？ |  |  |  |  |
| 13. 您常感到没有人很了解您吗？ |  |  |  |  |
| 14. 您常感到与别人隔开了吗？ |  |  |  |  |
| 15. 您常感到当您原意时就能找到伙伴吗？ |  |  |  |  |
| 16. 您常感到有人真正了解您吗？ |  |  |  |  |
| 17. 您常感到羞怯吗？ |  |  |  |  |
| 18. 您常感到人们虽然围着您但并不关心您吗？ |  |  |  |  |
| 19. 您常感到有人愿意与您交谈吗？ |  |  |  |  |
| 20. 您常感到有人值得您信赖吗？ |  |  |  |  |
