# Supplementary material for: The Influence of Health Literacy and Social Support on Loneliness Among Patients With Severe Mental Illness in Rural Southwest China
Source: Front Psychol. 2021 Sep 8;12:564666. doi: 10.3389/fpsyg.2021.564666 (PMC8455823; doi:10.3389/fpsyg.2021.564666)
Supplement: Supplementary file 1 [file Data_Sheet_1.ZIP › Supplementary File/Questionnaire——English.doc]

**Questionnaires**

**Part 1: Questions of socio-demographics**

1. Your gender：

1）Female	2）Male

2.Your age：

1）18-39	2）40-64	3）≥65

3.Your marital status：

1）Single	2）Married	3）Divorced or widowed

4.Your education level:

1）Less than elementary school	2）Elementary school

3）Middle school	4）High school and above

5.Your per capita annual income of household (¥):

1）＜5000	2）5,000-9,999	3）≥10,000

**Part 2: Questions on health literacy**

The following statement about mental health, you hold what kind of attitude?

|  | agree | disagree |
| --- | --- | --- |
| 1.Mental health is a component of health. | ① | ② |
| 2.Mental disorders are caused by incorrect thinking. | ① | ② |
| 3.Many people have mental problems but do not realize it. | ① | ② |
| 4.All mental disorders are caused by external stressors. | ① | ② |
| 5.Components of mental health include normal intelligence, stable mood, a positive attitude, quality interpersonal relationships, and adaptability. | ① | ② |
| 6.Most mental disorders cannot be cured. | ① | ② |
| 7.Psychological or psychiatric services should be sought if one suspects the presence of psychological problems or a mental disorder. | ① | ② |
| 8.Psychological problems can occur at almost any age. | ① | ② |
| 9.Mental disorders and psychological problems cannot be prevented. | ① | ② |
| 10.Even for severe mental disorders (e.g. schizophrenia), medications should be taken for a given period of time only; there is no need to take them for a long time. | ① | ② |
| 11.Positive attitudes, good interpersonal relationships and a healthy life style can help maintain mental health. | ① | ② |
| 12.Individuals with a family history of mental disorders are at a higher risk for psychological problems and mental disorders. | ① | ② |
| 13.Psychological problems in adolescents do not influence academic grades. | ① | ② |
| 14.Middle-aged or elderly individuals are unlikely to develop psychological problems and mental disorders. | ① | ② |
| 15.Individuals with a bad temperament are more likely to have mental problems. | ① | ② |
| 16. Mental problems or disorders may occur when an individual is under psychological stress or facing major life events (e.g. death of family members). | ① | ② |

**Part 3: Questions on social support**

1. How many close friends whom you can get support and help from do you have?

1） None	2）1~2	3）3~5	4）6 or above 6

2. Over the past year, you:

1） lived alone and were away from families

2）often changed the residence and lived with strangers for most of the time

3）lived with classmates, colleagues or friends

4）lived with families

3. You and your neighbors:

1） never care about each other
2） may care slightly when meet with difficulties
3） some neighbors very care about you
4） most of neighbors very care about you

4. You and your classmates/colleagues:

1） never care about each other
2） may care slightly when meet with difficulties
3） some classmates/colleagues very care about you
4） most of classmates/colleagues very care about you

5.1 You get support and care from your spouse (lover).

1） None	2）Very little	3）General	4）Fully support

5.2 You get support and care from your parents.

1）None	2）Very little	3）General	4）Fully support

5.3 You get support and care from your children.

1）None	2）Very little	3）General	4）Fully support

5.4 You get support and care from your siblings.

1）None	2）Very little	3）General	4）Fully support

5.5 You get support and care from other family members (such as sisters-in-law).

1）None	2）Very little	3）General	4）Fully support

6. In the past, when you were in a difficult situation, the source of the economic support and practical help was from:

1）None

2）The following (can select multiple items):

A. Spouse	B. Other family members	C. Friends	D. Relatives

E. Classmates/Colleagues	F. Company/School	G. Official or semi-official organizations

H. Unofficial organizations	I. Others

7. In the past, when you were in a difficult situation, the source of comfort and concern was from:

1）None

2）The following (can select multiple items):

A. Spouse	B. Other family members	C. Friends	D. Relatives

E. Classmates/Colleagues	F. Company/School	G. Official or semi-official organizations

H. Unofficial organizations	I. Others

8. When you are in a trouble, you talk to:

1）Nobody	2）1~2 people who is/are very close to you

3）friends if they ask you	4）people initiatively

9. When you are in trouble, you ask for help:

1）Never	2）Occasionally	3）sometimes	4）Frequently

10. You join group organizing activities (such as party organizations, religious organizations, trade unions, student unions, etc.).

1）Never	2）Occasionally	3）Frequently	4）Initiatively and actively

**Part 4: Questions on loneliness**

0 Never	1 Rarely	2 Sometimes	3 Often

| Please choose the answer that best suits your situation according to your daily feelings: | 0 | 1 | 2 | 3 |
| --- | --- | --- | --- | --- |
| 1. How often do you feel that you are “in tune” with the people around you? |  |  |  |  |
| 2. How often do you feel that you lack companionship? |  |  |  |  |
| 3. How often do you feel there is no one you can turn to? |  |  |  |  |
| 4. How often do you feel alone? |  |  |  |  |
| 5. How often do you feel part of a group of friend? |  |  |  |  |
| 6. How often do you feel that you have a lot in common with the people around you? |  |  |  |  |
| 7. How often do you feel that you are no longer close to anyone? |  |  |  |  |
| 8. How often do you feel your interests and ideas are not shared by those around you? |  |  |  |  |
| 9. How often do you feel outgoing and friendly? |  |  |  |  |
| 10. How often do you feel close to people? |  |  |  |  |
| 11. How often do you feel left out? |  |  |  |  |
| 12. How often do you feel that your relationships with others are not meaningful? |  |  |  |  |
| 13. How often do you feel that no one really knows you well? |  |  |  |  |
| 14. How often do you feel isolated from others? |  |  |  |  |
| 15. How often do you feel you can find companionship when you want it? |  |  |  |  |
| 16. How often do you feel there are people who really understand you? |  |  |  |  |
| 17. How often do you feel shy? |  |  |  |  |
| 18. How often do you feel that people are around you but not with you? |  |  |  |  |
| 19. How often do you feel that there are people you can talk to? |  |  |  |  |
| 20. How often do you feel that there are people you can turn to? |  |  |  |  |
